# NAD+ associated genes as potential biomarkers for predicting the prognosis of gastric cancer

**DOI:** 10.32604/or.2023.044618

**Published:** 2023-12-28

**Authors:** XIANGDONG SUN, HUIJUAN WEN, FAZHAN LI, IHTISHAM BUKHARI, FEIFEI REN, XIA XUE, PENGYUAN ZHENG, YANG MI

**Affiliations:** 1 Henan Key Laboratory for Helicobacter Pylori & Microbiota and GI Cancer, Marshall Medical Research Center, The Fifth Affiliated Hospital of Zhengzhou University, Zhengzhou, China; 2 Department of Gastroenterology, The Fifth Affiliated Hospital of Zhengzhou University, Zhengzhou, China

**Keywords:** NAD+, LncRNAs, Cancer, Cell infiltration, Prognosis, Tumor microenvironment

## Abstract

Nicotinamide adenine dinucleotide (NAD+) plays an essential role in cellular metabolism, mitochondrial homeostasis, inflammation, and senescence. However, the role of NAD+-regulated genes, including coding and long non-coding genes in cancer development is poorly understood. We constructed a prediction model based on the expression level of NAD+ metabolism-related genes (NMRGs). Furthermore, we validated the expression of NMRGs in gastric cancer (GC) tissues and cell lines; additionally, β-nicotinamide mononucleotide (NMN), a precursor of NAD+, was used to treat the GC cell lines to analyze its effects on the expression level of NMRGs lncRNAs and cellular proliferation, cell cycle, apoptosis, and senescence-associated secretory phenotype (SASP). A total of 13 NMRGs-related lncRNAs were selected to construct prognostic risk signatures, and patients with high-risk scores had a poor prognosis. Some immune checkpoint genes were upregulated in the high-risk group. In addition, cell cycle, epigenetics, and senescence were significantly downregulated in the high-risk group. Notably, we found that the levels of immune cell infiltration, including CD8 T cells, CD4 naïve T cells, CD4 memory-activated T cells, B memory cells, and naïve B cells, were significantly associated with risk scores. Furthermore, the treatment of NMN showed increased proliferation of AGS and MKN45 cells. In addition, the expression of SASP factors (IL6, IL8, IL10, TGF-β, and TNF-α) was significantly decreased after NMN treatment. We conclude that the lncRNAs associated with NAD+ metabolism can potentially be used as biomarkers for predicting clinical outcomes of GC patients.

## Introduction

Nicotinamide adenine dinucleotide (NAD+) is an essential coenzyme in cellular energy metabolism, including the citric acid (TCA) cycle, oxidative phosphorylation, and fatty acid metabolism [1,2]. It regulates enzymes, gene expression, DNA repair, cell cycle, mitochondrial homeostasis, inflammation, and cellular senescence [2,3]. NAD+ supplementation, such as β-nicotinamide mononucleotide (NMN), prevents senescence in stem cells [4,5], and its reduced level has been detected in an elderly person with neurodegenerative disorders [6]. It is widely recognized that NAD+ depletion causes mitochondrial dysfunction, leading to reduced ATP production and low metabolism, as NAD+ is crucial for mitochondrial function [2,7]. In addition, NAD+ contributes significantly to cellular energy metabolism as a substrate for several enzymes and plays an essential role in DNA damage repair and epigenetic regulation [8,9]. Restoring NAD+ levels can delay senility, improve health, and enhance immunity [10,11].

Cancer cells constantly reprogram their metabolism to support the high proliferation rates, such as the Warburg effect of aerobic glycolysis [12–14], a critical metabolic hallmark in cancer. NAD+ levels and the NAD+/NADH (nicotinamide adenine dinucleotide) ratio are much higher in cancer cells than in normal cells, suggesting the vital role of NAD+ in cancer cell metabolism [12]. It has also been reported that NAD+ can strongly induce highly pro-inflammatory senescence-associated secretory phenotype (SASP) and accelerate cancer progression [10]. Several studies have shown that targeting NAD+ synthesis induces cancer cell cytotoxicity upon NAMPT inhibitor *in vitro* and *in vivo* [11,13–17]. Although previous clinical trials of NAD+ inhibitors showed no effect on remission of advanced solid tumor [18–22], NAD+ is still an intriguing target for cancer therapy and a better understanding of its role in cancer may show some clinical success.

Gastric cancer (GC) ranked the fourth most common cause of cancer-associated deaths globally in 2020 due to its high incidence rate [23]. To date, chemotherapeutics and targeted therapeutics are the traditional treatments for GC but have serious side effects for patients [24]. Knockdown of NAMPT inhibits NAD+, which slows the progression of GC cells by reducing the metabolic rate, while induction of NAD+ by β-nicotinamide mononucleotide (NMN) can reverse cell growth [14]. However, the underlying mechanisms remain unclear, and we aimed to determine the effect of NAD+ induction in GC. In the present study, we performed bioinformatic analyses to identify the coding and long non-coding genes associated with NAD+ metabolism in GC samples obtained from TCGA (The Cancer Genome Atlas https://portal.gdc.cancer.gov/cart). We analyzed the survival, prognosis, and immune regulation of GC patients based on the risk score calculated in the model of NAD+ metabolism related genes (NMRGs) and related lncRNAs. In addition, we collected GC tissues and quantified the expression levels of the NAD+ metabolism related genes and lncRNAs. To determine NMRGs and lncRNAs function, we treated GC cells with NMN (an inducer of NAD+) to analyze its effect on cell proliferation, cell cycle, apoptosis, and other related phenotypes.

## Materials and Methods

### Data collection

The TCGA-STAD (stomach adenocarcinoma, STAD) datasets, including gene expression profiles, DNA mutations, and clinical information, were obtained from GDC (https://portal.gdc.cancer.gov/cart) [25]. Transcriptomic data from 375 GC tissues and 32 normal tissues were analyzed. Clinicopathological data from 317 GC patients were selected for the OS and risk score analysis.

### NAD+ metabolism genes mutation and expression

NMRGs were obtained from the Kyoto Encyclopedia of Genes and Genomes (KEGG) database (Pathway: hsa00760; Permission Ref: 230570) [26–28] and the Reactome database (Reactome: R-HSA-196807) [29]. We identified 42 overlapping NMRGs in the TCGA-STAD datasets. The somatic mutation profiles of the NMRGs were further analyzed using the R package “maftools” [30]. The difference in expression of NAD+ metabolism genes was quintile normalized (R package: “edgeR”) and analyzed by the Wilcox test [31]. The expression level of NMRGs was extracted from each case (|logFC| > 1 and *p* < 0.05) for further analysis.

### The selection of NAD+ metabolism-associated related lncRNAs

The different expressions of the lncRNAs were individuated using Strawberry Perl and the package “limma” [32]. The correlation between NMRGs and lncRNAs was determined by Pearson correlation analysis (threshold; cor > 0.4 and *p* < 0.001). Univariate Cox proportional hazard regression analysis was used to screen for lncRNAs associated with survival in GC patients and these lncRNAs were further used to estimate the risk score.

### Construction and validation of NMRGs related lncRNAs risk signature

LASSO regression was utilized to identify the most crucial lncRNAs. This analysis was repeated 1000 times to cross-validate and pinpoint the best lambda value for the hub lncRNAs. The risk scores were then determined based on those optimal candidate hub lncRNAs, utilizing the LASSO coefficient, expressed as Risk score = Σ βi × lncRNAi, where βi represents the coefficient of the i-th lncRNA and lncRNAi is the expression level of the i-th lncRNA [33]. GC tissues were categorized into groups with high and low-risk scores groups determined by the median of the risk score. The overall survival rate was then determined using R packages “survival” and “survminer” [34]. The receiver operating characteristic curve (ROC) and Area Under Curve (AUC) were plotted using the R package “survival ROC” to demonstrate the accuracy of the prognostic model [35]. According to the risk score, subgroups, including low- and high-risk groups, were established by applying surv_cutpoint and surv_categorize of the “survminer” package (ninprop = 0.3). In order to determine the prognostic value of the constructed model, we analyzed the relationship between the model and clinical factors by using the chi-square test. A nomogram was created by the risk score, age, gender, and tumor stage to predict 1-, 2-, and 3-year overall survival. Correction curves, based on the Hosmer–Leme test, were used to evaluate the consistency of the prediction outcomes with practical results.

### The immune infiltration in TME and immune checkpoints

Immune-cell factors were analyzed between the risk groups, and the immune cell infiltration status among the GC patients was calculated by the package “CIBERSORT” [36]. The immune scores, stromal scores, and tumor purity were estimated utilizing the “ESTIMATE” package in the R software [37]. The correlation between the risk score and estimation score was analyzed using the “cor” package and a scatter plot showing the correlation was created with “ggplot2” package [38]. Then, we chose some immune checkpoint genes (Suppl. Table S1) to detect immunotherapy responses in the risk groups.

### Gene enrichment analysis

The different expression of genes between high and low-risk groups was analyzed using edgeR normalization and the Wilcox test. The different expression genes are revealed as the volcano plot and the heatmap (*p* < 0.05, |logFC| > 2). The Gene Ontology (GO) terms comprised Biological Process (BP), Cellular Component (CC), and Molecular Function (MF) categories. Enrichment analyses of these terms utilized “clusterProfiler” and “ggplot2” packages within R software (version 4.2.0) [39,40]. The GSEA was performed by the package ReactomePA [29] and analyzed the pathways related to NAD+-based DNA damage repair, epigenetics, and cell senescence by the “enrichplot” package.

### The expression of NMRGs and lncRNAs in the GC tissues

The GC tissues were collected from the Fifth Affiliated Hospital of Zhengzhou University, and the Ethics Committee of the same Hospital (KY2020029) approved the protocols used in our study. All study participants received a verbal explanation regarding the project’s details, followed by obtaining informed consent. All the sampling and experimental procedures were conducted with strict adherence to the guidelines of the Helsinki Declaration 1964 and its latest amendments. The total RNAs of 19 GC tissues and cell lines were extracted with TRIzol (#15596026, Invitrogen). The cDNA was reverse-transcribed by the First Strand cDNA Synthesis Kit (#FSK-101, TOYOBO). The real-time polymerase chain reaction (PCR) primers are listed in the Suppl. Table S2. The PCR was performed on the Roche LightCycler480 system, and the quality was determined using GAPDH as a reference through the 2^−ΔΔCt^ approach [41].

### Cell culture

Human GC cell lines (AGS, MGC803, MKN45, and SGC 7901) were purchased from the Institute of Biochemistry and Cell Biology of the Chinese Academy of Sciences (Shanghai, China). AGS cells were cultured in the DMEM/F12 medium (11320033, Gibco, USA). All other cell lines were cultured in the RPMI1640 medium (#01-106-1A, Bioind), with 10% fetal bovine serum (FBS, #04-001-1-1A, Bioind) and 1% penicillin-streptomycin (#C0222, Beyotime), at 37°C in a humidified incubator with constant supply of 5% CO_2_.

### Cell proliferation assay

For the cell proliferation assay, a Cell Counting Kit-8 (CCK-8; #CK04, Dojindo Laboratories, Kumamoto, Japan) was used to determine cell viability. Briefly, the cells were seeded in the 96-well plates and treated with NMN (#N3501, Sigma-Aldrich, USA) at different concentrations (0.5, 1.0, 2.0, 3.0, and 4.0 μM). After culturing for the indicated times (24, 48, 72, and 96 h), 10 ul CCK-8 reagent was added to cells for 1 h. Absorbance was measured at 450 nm with a Microplate Reader (iMark, Bio-Rad, USA). For each treatment group, samples were assessed in triplicate, and the experiments were independently repeated thrice.

### Cell cycle assay

For cell cycle analysis, cell-cycle distribution was determined with the cell cycle kit (#KGA512, Keygen, China) by flow cytometry (CytoFlex, Beckman, USA). Briefly, the cells were seeded in the 6-well plates, and different concentrations (0.5, 1.0, 2.0, 3.0, and 4.0 μM) of NMN were added. After culturing for 48 h, cells were digested with trypsine. The single cells were fixed with 75% ethanol for 2 h at 4°C. After fixing, the cells were washed thrice with PBS and centrifuged at 500 × g. The PI/RNase solution was added to the cells, stained in the dark at room temperature for 30 min, and detected by flow cytometry at the FL3 (Ex = 488 nm, Em ≥ 630 nm) channel. The treatments were assessed in triplicate, and the experiment was independently repeated three times. After flow cytometry, data were analyzed with ModfitLT software.

### Cell apoptosis assays

The cell apoptosis assay was performed using the Annexin V-FITC/PI kit (#KGA108, Keygen, China) and analyzed via flow cytometry (CytoFlex, Beckman, USA). Briefly, the cells were seeded into the 6-well plates and subjected to various concentrations (0.5, 1.0, 2.0, 3.0, and 4.0 μM) of NMN. After culturing for 48 h, cells were digested with trypsin (without EDTA). The cells were washed three times with PBS prior to adding 500 μl binding buffer. 5 μl Annexin V-FITC and 5 μl Propidium Iodide were added and stained for 10 min in the dark at room temperature. The stained cells were detected using flow cytometry at FL1 (Ex = 488 nm, Em = 530 nm) and FL3 (Ex = 488 nm, Em ≥ 630 nm) channels in 1 h. The experiments were performed thrice independently. After flow cytometry, the FlowJo software was used to analyze the data.

### Analysis of senescence-associated secretory phenotype (SASP)

To analyze SASP, the expression levels of IL-6, IL-8, IL-10, TGF-β, and TNF-α mRNA were detected by real-time PCR. The AGS cell line was treated with different concentrations of NMN (0.5, 1.0, 1.5, and 2.0 μM) for 48 h, and the whole RNA was extracted, and cDNA was synthesized. The real-time PCR primers are shown in Suppl. Table S2. The real-time PCR was performed on the Roche LightCycler480 system. The relative quality of expression was calculated using 2^−ΔΔCt^ [41].

### Statistical analysis

Statistical analysis was carried out using R (version 4.2.0). To determine the significance of differences between the two groups, we conducted a two-tailed Student’s *t*-test. For evaluating the effectiveness of differences among more than two groups of independent samples, we used One-way ANOVA. Additionally, to determine the correlation between two variables, we performed Pearson’s correlation analysis. The survival rate was analyzed using the Kaplan-Meier method.

## Results

### The landscape of NAD+ metabolism genes in GC

**Figure 1:**
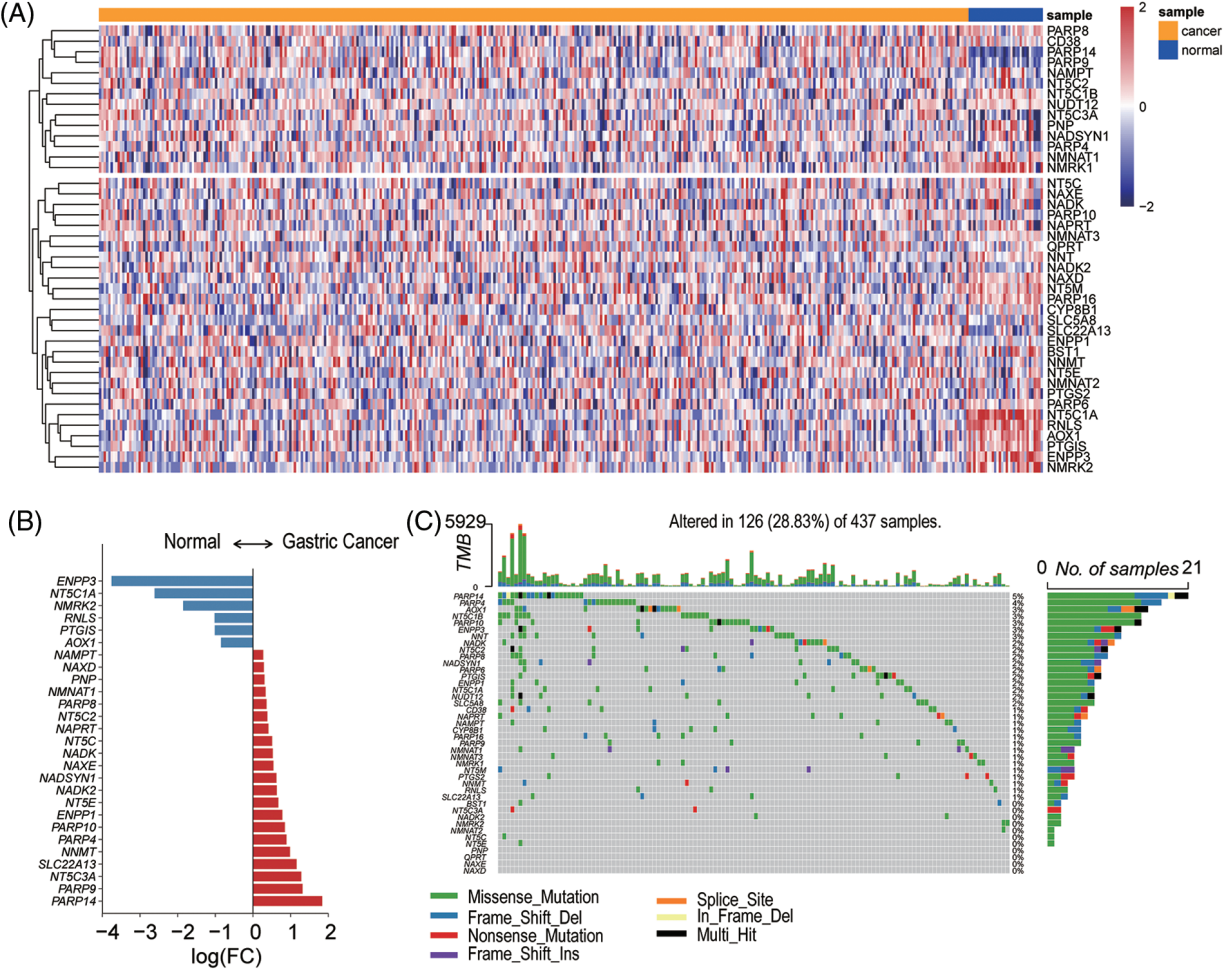
The expression and genomic alterations of NAD+ metabolism-related genes in gastric cancer. (A) The heatmap of NMRGs in GC patients (blue, normal tissues, orange, cancer tissues) normal tissue and GC tissues. (B) Differential expression of NMRGs between cancer and normal tissues (*p* < 0.05). The data shown were based on the TCGA data portal. (C) The landscape of NMRGs mutations in GC samples.

We collated the clinical characteristics of the TCGA-STAD samples (Table 1) and observed the expression pattern of NMRGs in GC tissues. The mRNA expression levels of 27 genes related to NMRGs showed significant alterations in GC (*p* < 0.05) (Figs. 1A and 1B). Among them, PARP14, PARP9, NT5C3A, and SLC22A13 were upregulated (logFC > 1), while PTGIS, RNLS, NMRK2, NT5C1A, and ENPP3 showed downregulated (logFC < −1) (Fig. 1B). When considering genomic alterations, we observed that the mutations of the NMRGs, such as PARP14, PARP4, AOX1, NT5C1B, PARP10 and ENPP3, showed a higher mutation burden, but the percentages of mutations in these genes were not so high (Fig. 1C).

**Table 1:** The clinical characteristics of TCGA-STAD gastric cancer patients

Clinical characteristics	Risk	Total
Age	<=65	164 (43.73%)
	>65	207 (55.2%)
	Unknown	4 (1.07%)
Gender	FEMALE	134 (35.7%)
	MALE	241 (64.3%)
Grade	G1–2	147 (39.2%)
	G3	219 (58.4%)
	Unknown	9 (2.4%)
Stage	Stages I–II	164 (43.73%)
	Stages III–IV	188 (50.13%)
	Unknown	23 (6.14%)
T	T1–2	99 (26.4%)
	T3–4	268 (71.47%)
	Unknown	8 (2.13%)
M	M0	330 (88%)
	M1	25 (6.67%)
	Unknown	20 (5.33%)
N	N0	111 (29.6%)
	N1–3	246 (65.6%)
	Unknown	18 (4.8%)

### NMN metabolism genes-related lncRNAs in GC patients

**Figure 2:**
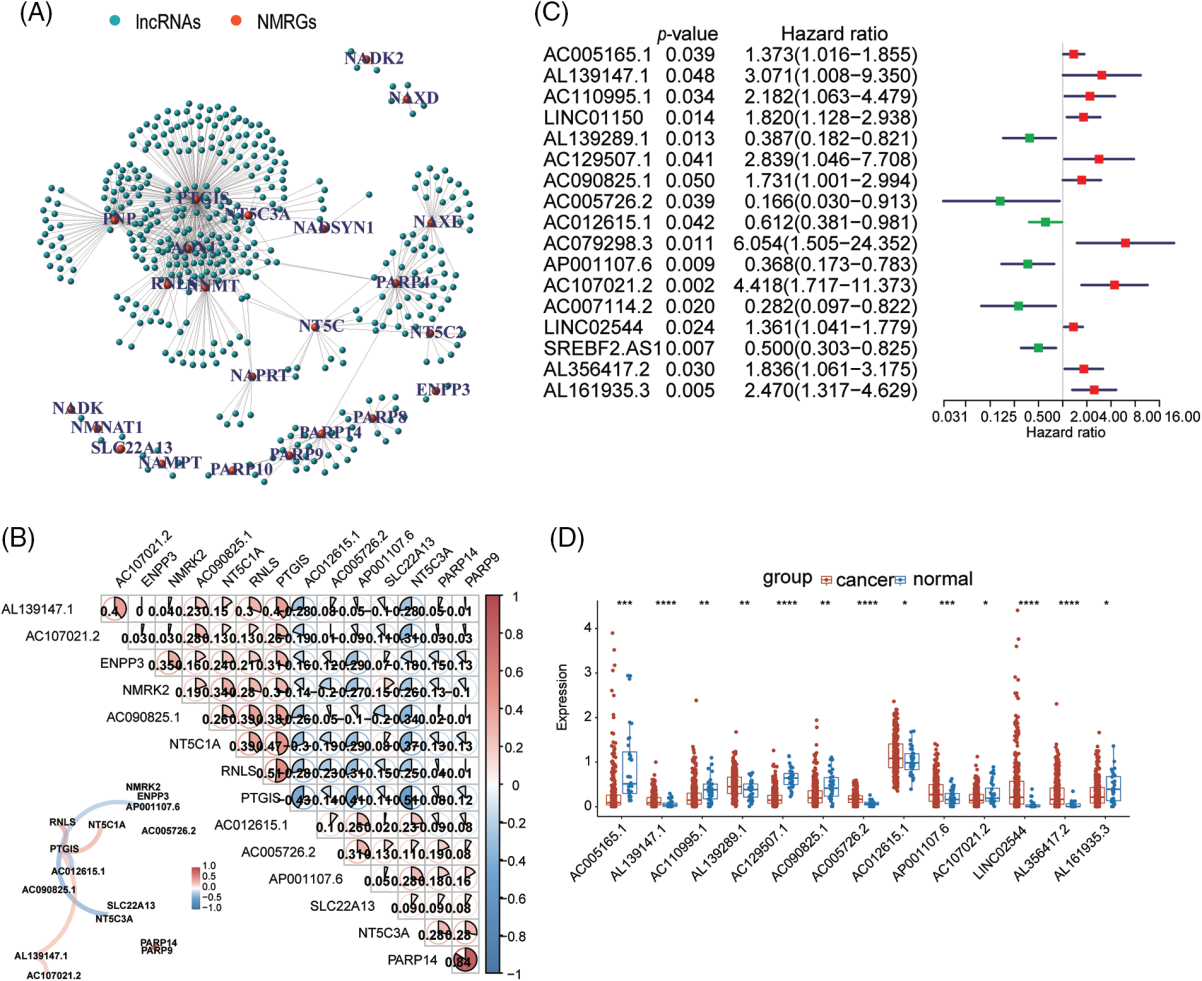
Identification of NMRGs related lncRNAs in GC patients. (A) The network between NMRGs and lncRNAs (cor > 0.4 and *p* < 0.05). Red points: NMRGs, green points: lncRNAs. (B) The correlation matrix and network of NMRGs and lncRNAs. Red: positive relation, blue: negative relation. (C) The univariate Cox regression analysis of NMRGs related lncRNAs. Red square: risk factors, green square: good factors. (D) The expression of NMRGs-related lncRNAs between GC tissues and normal tissues. *p < 0.05, **p < 0.01, ***p < 0.001, ****p < 0.0001.

According to the expression of NMRGs, we constructed a multi-cox model (Suppl. Fig. S1A) and classified GC patients into high-risk and low-risk groups. The results of Kaplan-Meier survival analysis indicated that patients with a high-risk score experienced an adverse survival outcome (Suppl. Fig. S1B, *p* < 0.0001). Additionally, a significant difference was observed in survival ratio (Suppl. Fig. S1B). The AUC was 0.447, which is not a good survival prediction model (Suppl. Fig. S1C). According to the expression of NMRGs and lncRNAs between normal and tumor tissues, we finally identified 413 NMRGs-related lncRNAs (|cor| > 0.4 and *p* < 0.001). The network figure and data between NMRGs and lncRNAs are shown in Figs. 2A, 2B, and Suppl. Table S3. Based on the univariate Cox regression analysis, we identified 17 lncRNAs related to NMRGs that significantly correlate with overall survival (OS) (*p* < 0.05) (Fig. 2C). Besides, compared with the normal tissues, seven lncRNAs (AL139147.1, AL139289.1, AC005726.2, AC012615.1, AP001107.6, LINC02544, AL356417.2) were up-regulated while six lncRNAs (AC005165.1, AC110995.1, AC129507.1, AC090825.1, AC107021.2, AL161935.3) were down-regulated in GC tissues (Fig. 2D).

### Construction and verification of the prediction model

**Figure 3:**
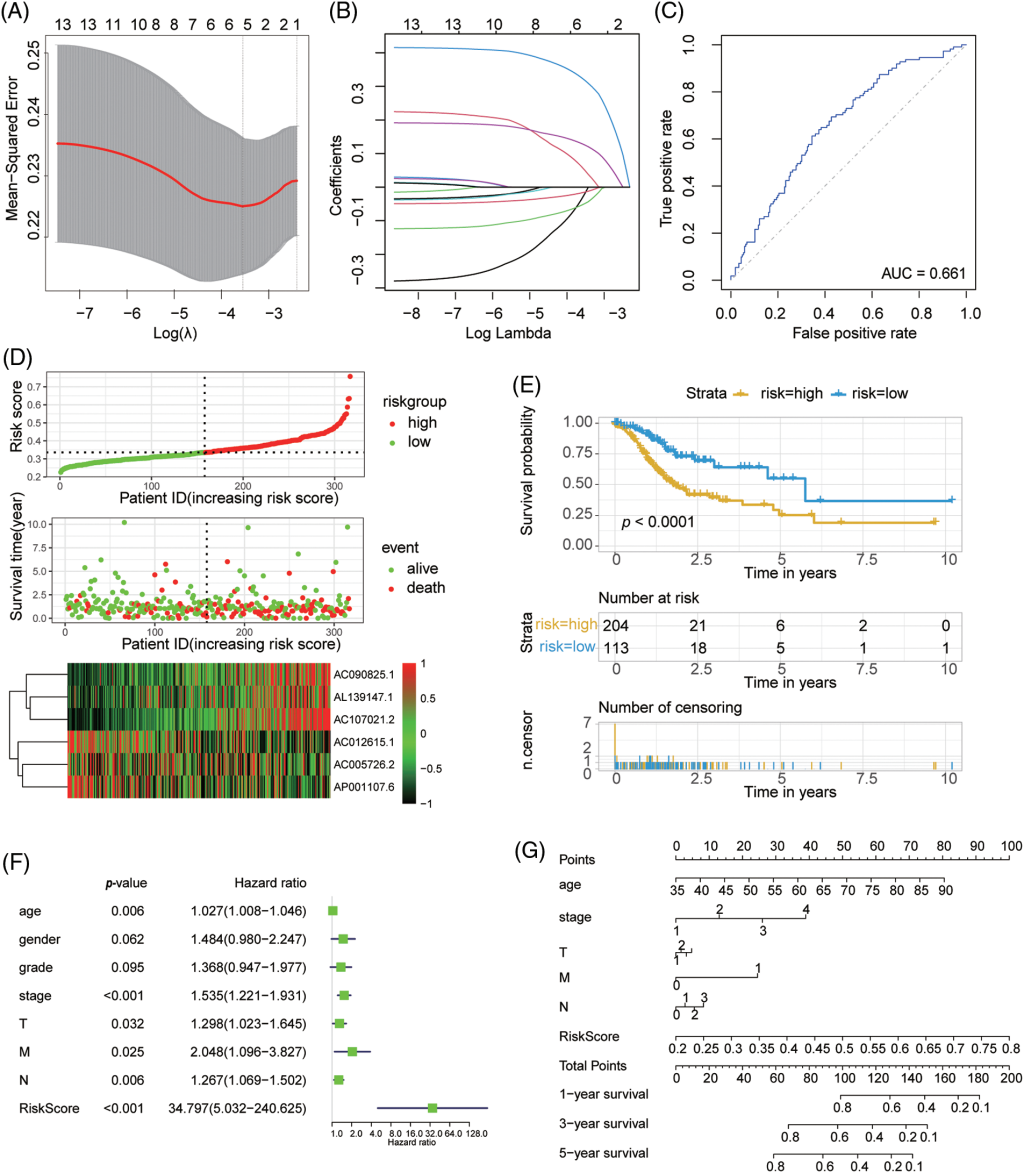
The NMRGs related lncRNAs prognosis model in GC. (A) LASSO coefficient profile of NMRGs associated lncRNAs. (B) 10-fold cross-validation for variable selection in the LASSO model. (C) ROC curve analysis to evaluate the performance of the Cox model of NMRGs relative lncRNAs. (D) Distribution of risk scores, survival status of patients, and heatmap of NMRGs relative lncRNAs expression. (E) Kaplan-Meier survival curves of OS (survival probability) of GC patients between low- and high-risk groups, based on the multi-Cox model of NMRGs related lncRNAs. (F) Multi-Cox regression survival analysis in GC patients. (G) The OS prediction nomogram based on the multi-Cox regression survival analysis model.

We performed the Lasso regression analysis to screen the aforementioned lncRNAs (Figs. 3A and 3B). We identified six lncRNAs with the minimum likelihood of deviance corresponding to the first-rank value of Log (λ). The model’s AUC was plotted using the calculation procedure (AUC = 0.661, Fig. 3C). We calculated risk score with the formula: risk score = AL139147.1 × (0.416) + AC107021.2 × (0.3119) + AC090825.1 × (0.1218) + AC005726.2 × (−0.0.0062) + AC012615.1 × (−0.0130) + AP001107.6 × (−0.0451). The risk score formula was used to compare the distribution of risk score, the survival status, survival time, and the corresponding expression standards of these lncRNAs of patients between low and high risk groups. We found that patients in the high-risk group had a poor prognosis (Figs. 3D and 3E). The conventional clinicopathological characteristics, age, gender, grade, stage, T (tumor), M (metastasis), and N (node) were also recorded (Fig. 3F). Patients in the high risk group had significantly worse overall survival than the low group at the age, gender, grade, stage, T, M and N (Suppl. Fig. S2). Based three independent prognostic factors, risk score, age, and TNM stage (*p* < 0.05), we built a nomogram to predict the 1-, 2-, and 3-year OS of GC patients (Fig. 3G). We also utilized the 1-, 2-, and 3-year calibration plots which showed that the nomogram had a significant association with the prediction of 1-, 2-, and 3-year OS (Suppl. Fig. S3).

### The investigation of immunity factors and clinical treatment in risk groups

**Figure 4:**
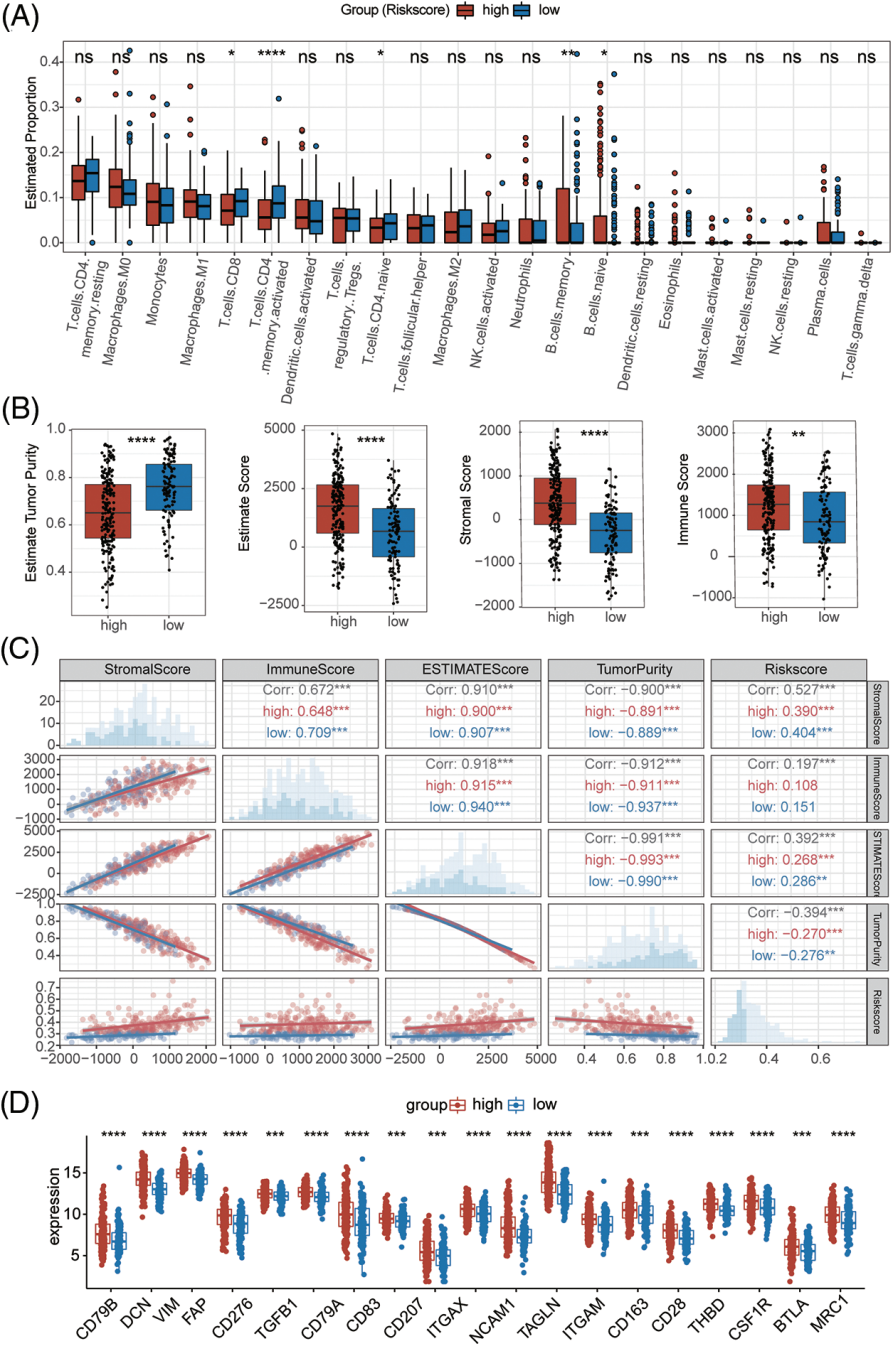
Immune profile alterations by the prognostic signature. (A) Peripheral infiltrating levels in the high-risk and low-risk groups using CIBERSORT algorithms. (B) The immune-related scores between high-risk and low-risk groups using ESTIMATE. (C) The correlation between the risk scores and immune-related scores. (D) The differential expression of checkpoint genes between high-risk and low-risk groups. *p < 0.05, **p < 0.01, ***p < 0.001, ****p < 0.0001.

By quantifying the degree of immune cell infiltration in the samples, we found that CD8+ T cells, memory-activated CD4+ T cells, and CD4+ naïve T cells were associated with the low-risk group. Memory B cells and naïve B cells were associated with the high-risk group (Fig. 4A). The high-risk group had a higher estimation score, stromal score, and immune score, indicating a different TME than the low-risk group (Figs. 4B and 4C). Some immune checkpoints (CD78A, CD 78B, DCN, VIM, CD276, CD207, TGFB1, CD83, ITGAX, ITGAM, NCAM1, CD163, CD28, TAGLN, BTLA, MRC1, THBD, CSF1R) also showed better activation in the high-risk group (Fig. 4D, Suppl. Table S4). These results suggested that the high-risk group not only had higher NAD+ metabolic activity but also higher levels of immune cell infiltration in the tumor tissues.

### Functional analyses of NMRGs

**Figure 5:**
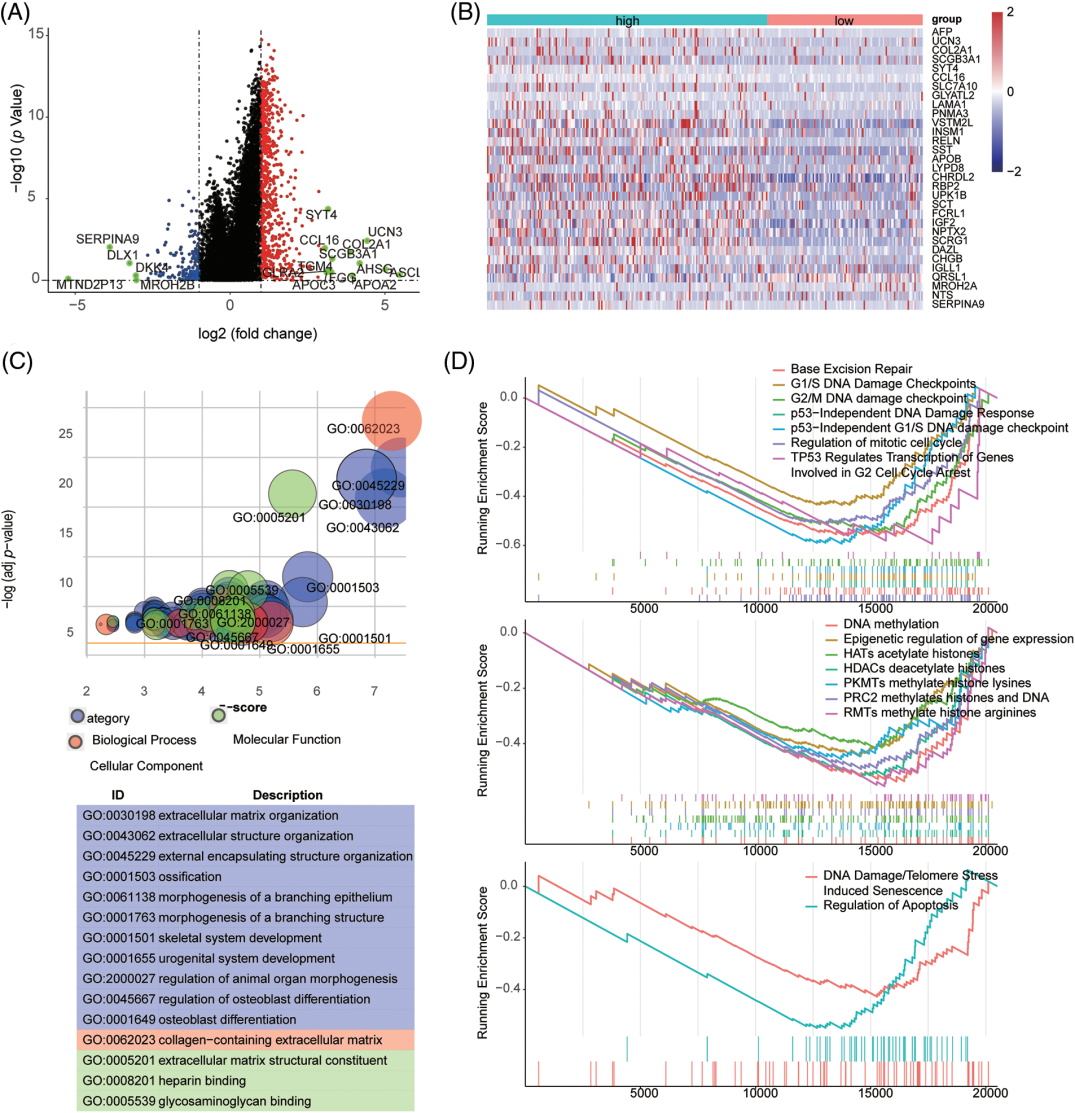
Functional annotation and GSEA with the prognostic signature. (A) The volcano of differential expression genes (DEGs) between the low-risk and high-risk groups. Red points, upregulated genes, blue: downregulate genes, green points: |logFC| > 3. (B) The heatmap of differential expression genes between the low-risk and high-risk groups. (C) GO analysis of DEGs. (D) GSEA showed the NAD+-related pathways. These pathways were enriched in the low-risk group.

To investigate differences in biological functions and pathways, we analyzed the expression of the different genes between risk groups. The expression of the genes |logFC| > 3 and *p* < 0.05 was plotted as a volcano plot and heatmap (Figs. 5A and 5B). GO enrichment showed that these differential genes have a potential role in cellular development (Fig. 5C). Meanwhile, these were primarily enriched pathways in the low-risk group (*p* < 0.05 and NES < 0, Fig. 5D). The results suggest that high-risk group may develop or provoke cancer reprogramming due to inhibition of DNA damage repair, alteration in the methylation pattern of the proteins, or inhibition of the cell senescence or apoptosis.

### NMRGs and related lncRNAs expression in GC tissue

**Figure 6:**
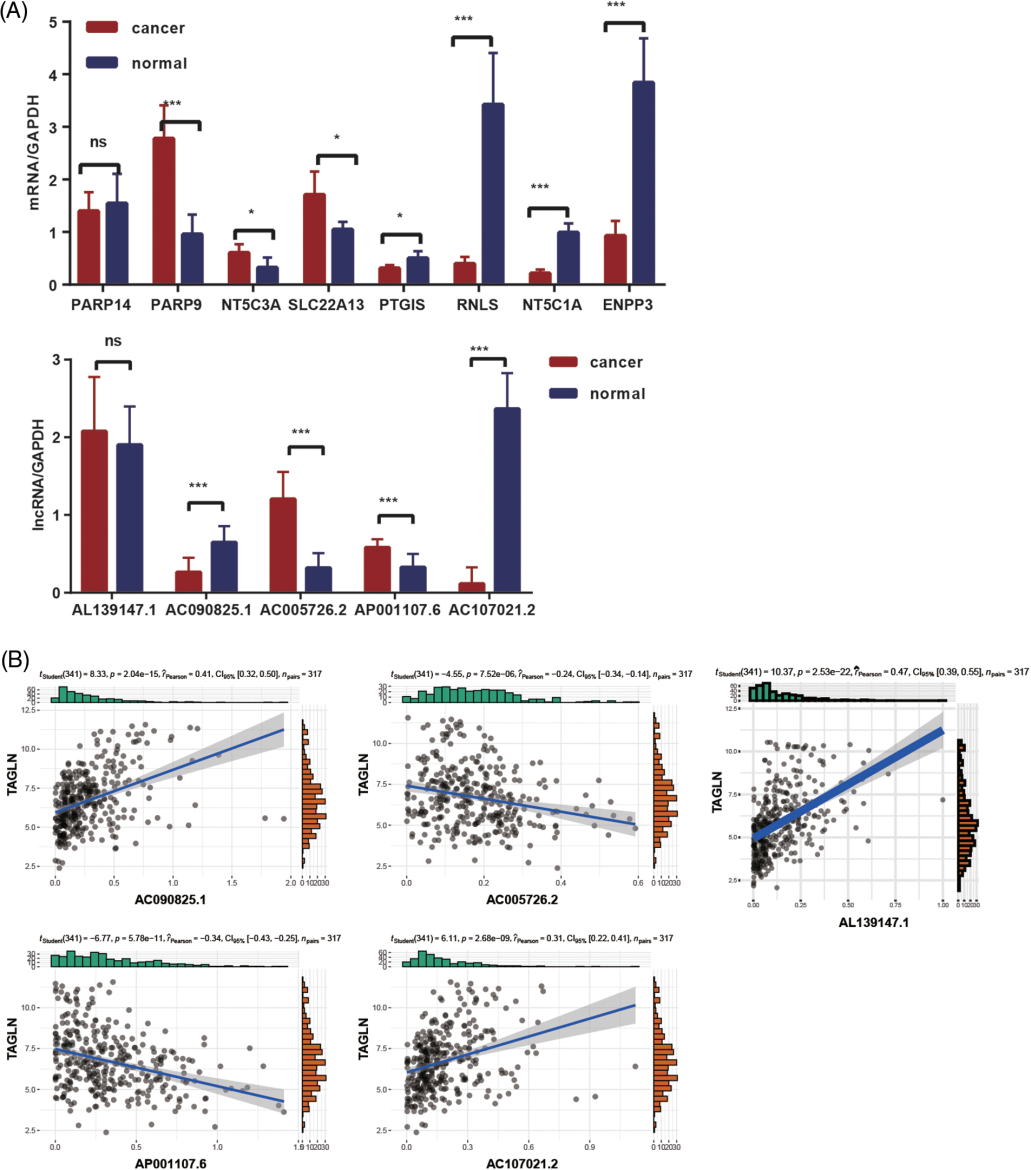
NMRGs and lncRNAs’ expression in GC tissues. (A) The expression of NMRGs and lncRNAs in GC and normal tissues. (B) The correlation of TAGLN and lncRNAs. The data was based on the TCGA data portal. *p < 0.05, ***p < 0.001.

The expression of the selected NMRGs and lncRNAs in clinical samples was analyzed by real-time PCR. A similar pattern of expression was reported for genes (PARP9, NT5C3A, SLC22A13, PTGIS, RNLS, NT5C1A, ENPP3) and lncRNAs, (AL139147.1, AC090825.1, AC005726.2, AP001107.6, and AC107021.2) as shown in the TCGA-STAD data analysis (Fig. 6A). Compared with the normal tissues, the lncRNAs AC 090825.1 and AC107021.2 showed lower expression in the GC tissues (*p* < 0.001), but the AC005726.2 and AP001107.6 had higher expression in the GC tissues (*p* < 0.001). The association of these five lncRNAs with immune checkpoints was further analyzed (Suppl. Table S5). We also found that lncRNAs (AL139147.1, AC090825.1, AC005726.2, AP001107.6, and AC107021.2) were strongly associated with immune response checkpoints genes TAGLN (Fig. 6B). AC005726.2 and AP001107.6 had a negative relation with TAGLN (Pearson = −0.239 and −0.344, respectively). AL139147.1, AC090825.1 and AC107021.2 were positively related to TAGLN (Pearson = 0.47, 0.411 and 0.314, respectively). The relative expression levels of NAD+ metabolism-related genes and lncRNAs in the tumor tissues were consistent with what we detected in the TCGA database.

### NMN reduces aging and enhances the proliferation of GC cells

**Figure 7:**
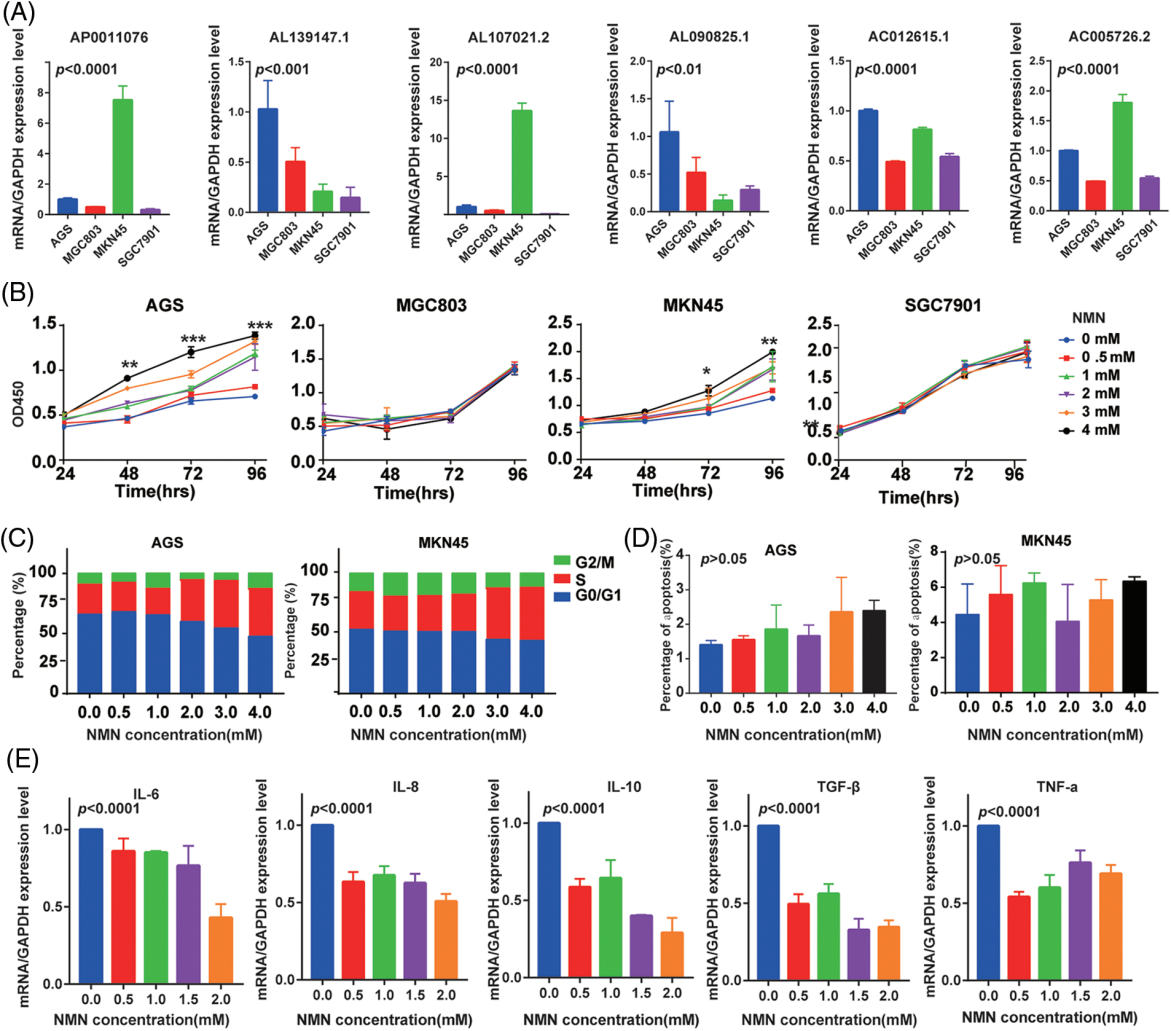
The effects of NMN on the GC cell lines. (A) The expression of lncRNAs in GC cell lines. (B) The proliferation of GC cell lines after treatment with different concentrations of NMN. (C) The cell cycles of GC cell lines after NMN treatment. (D) The cell apoptosis of GC cell lines under different concentrations of NMN. (E) The SASP factors mRNA expression of GC cell lines. *p < 0.05, **p < 0.01, ***p < 0.001.

The expression levels of lncRNAs in the gastric cell lines were detected by real-time PCR, and we found that lncRNAs had different expression levels among the gastric cell lines (Fig. 7A, *p* < 0.001). To analyze the effect of NAD+ on cell proliferation in GC, we treated AGS and MKN45 cells with various concentrations of NMN. We found that the proliferation of these cells was significantly improved by NMN (Fig. 7B, *p* < 0.001). The percentage of S-phase cells was higher than in the control group (Fig. 7C and Suppl. Fig. S4A, *p* < 0.001). We also found that the apoptosis rate in GC cells showed no significant difference with or without NMN (Fig. 7D and Suppl. Fig. S4B, *p* > 0.05). Furthermore, we analyzed the role of NMN in cellular senescence and found that the SASP factors, the mRNA levels of inflammatory factors, such as IL-6, IL-8, IL-10, TGF-β, and TNF-α (Fig. 7E, *p* < 0.001) were significantly decreased under NMN treatment. These results suggested that NMN treatment could improve GC development and inhibit cellular senescence.

## Discussion

NAD+ is one of the essential factors in the metabolic redox reaction [2] and serves as a substrate for enzymes involved in many pathways of cellular metabolism [6,42]. NAD+ is commonly synthesized from tryptophan, nicotinic acid, nicotinamide, or nicotinamide riboside [42–44]. However, the underlying molecular pathways of NAD+ synthesis and metabolism in GC remain not to be fully elucidated.

The impairment of NAD+ function could lead to abnormalities in neuronal calcium homeostasis and DNA repair ability [45–47] and DNA methylation [8,48]. NAD+ supplementation has become an important regulator of several cancers [49] and various strategies to target NAD+ metabolism in cancer have been used in clinical trials [49]. We investigated the NMRGs using RNA sequence data of GC patients obtained from TCGA. 27 genes explicitly related to NAD+ metabolism in GC were filtered out, and the top 4 up-regulated (PARP14, PARP9, NT5C3A, and SLC22A13) and top 5 down-regulated (PTGIS, RNLS, NMRK2, NT5C1A, and ENPP3) genes were selected for further analysis. The higher expression of PARPs causes double-strands breaks (DSBs) or other types of DNA damaged DNA to be repaired [50–52]. The proteins encoded by PARP14 and PARP9 are DNA damage repair enzymes that act as tumor suppressors by consuming NAD+ to stabilize cell metabolism and prevent genomic instability from containing potential oncogenic mutations [53,54]. It is well known that the higher mutational burden of tumors leads to alterations in immune functions, resulting in poor survival and prognosis [55–58]. The ratio of NMRGs mutation in GC patients was significantly low, suggesting that a high conservation of the NAD+ metabolism related genes.

Recently, lncRNAs have been identified as critical factors in cancer development [59,60]. In this study, firstly we found that the prediction model of NMRGs was not so good (AUC = 0.447). Then, we reported that NMRGs associated lncRNAs expression level may predict the prognosis of GC (AUC = 0.661). In our prognostic model, AC107021.2 was one of six hub indicator lncRNAs associated with the hypoxia-pathway [61]. The HIF1 signaling pathway often plays an important role in gastric carcinogenesis. According to the prediction model, GSEA enrichment results indicate that the high-risk group can inhibit DNA damage repair, DNA methylation, cell cycle arrest, apoptosis, and cellular senescence, which always prevent cellular carcinogenesis. We speculate that the NAD+ metabolism-related lncRNAs may also be involved in the immune regulation of the tumor microenvironment. The lncRNAs in the low-risk group showed an association with CD8+ T cells, memory-activated CD4+ T cells, and CD4+ naïve T cells. Meanwhile, high-risk patients showed activated immune checkpoints and high immune and stromal scores. Activating immune checkpoints is crucial for recruiting more immune cells and blocking immune checkpoints, which can inhibit tumor growth [62–64]. PD-1 and PD-L1 inhibitors, combined with regular chemotherapy, had a remarkable effect on the tumor growth [65–67]. In our study, some lncRNAs, such as the AL139147.1, AC090825.1, AC005726.2, AP001107.6, and AC107021.2, were related to the TAGLN, which could promote the metastasis of the advanced colorectal cancer [68,69] or as potential molecular targets to prevent colorectal cancer progression [70,71]. TAGLN expression was upregulated in GC-associated fibroblasts (CAFs), which promote GC cell migration and invasion [68]. NAD+ not only promotes gastric cancer progression but also promotes immune cell infiltration into tumors. It also suggests that modulation of NAD+ can inhibit inflammation and aging; therefore, it may not be appropriate for cancer patients.

The vital role of NAD+ in both energy metabolism and pathway regulation makes it critical for the growth and proliferation of several cancer cells [70]. *In vitro*, the expression of lncRNAs in AGS and MKN45 means their high-risk cell lines. The NMN supplementation could promote cancer cell proliferation. NMN could be rapidly absorbed and converted to NAD+ *in vivo* [72]. NMN supplementation has increased NAD+ biosynthesis, suppressed age-related inflammation, increased insulin sensitivity, improved mitochondrial function, etc. [73]. These results suggest that NMN could increase cancer metastases and development. This study outlines the role of NMN in cancer metabolism and the need to personalize its use in certain patients.

Although the lncRNAs and NMRGs identified in our research could potentially be used in clinical practice, our study has some limitations, such as the lack of clinical trials of these or other lncRNAs. Although checkpoint activation was significantly high in high-risk groups, we could not compare the expression of immune checkpoints and their inhibitors in the patients due to the unavailability of a large number of samples.

## Conclusions

NAD+ metabolism-derived lncRNAs may be promising biomarkers for predicting clinical outcomes and ultimately facilitating the precise management of GC patients. The different metabolic status of NAD+ determines the use of NNM supplements in GC. Therefore, the present study preliminarily revealed the mechanisms and correlations between NAD+ metabolism, lncRNAs, immunity, and GC at the genomic and molecular level. NMRGs related lncRNAs can be used as biomarkers for the diagnosis, therapeutics, and prognosis of gastric cancer patients.

## Supplementary Materials

Figure S1 NAD+ metabolism-related genes prognostic model A. The univariate Cox regression analysis of NMRGs. B. The Kaplan-Meier survival curves of OS (survival probability) of GC patients between low- and high- groups, which are based on the multi-Cox model of NMRGs. C. Receiver operating characteristic curve (ROC) analysis to evaluate the performance of the multi-Cox model of NMRGs.

Figure S2 Kaplan-Meier survival curves OS (survival probability) between low- and high-groups. The prognostic value is stratified by old patients (age<= 65 and age= 65), gender (male and female), grade (grade1/2 and grade3), stage (stage1/2 and stage3/4), T (T1/2 and T3/4), M (M0 and M1), and N (N0/1 and N2/3/4).

Figure S3 The calibration curve for the nomogram is based on the Multi-Cox regression survival analysis model.

Figure S4 The effects of NMN on the GC cell cycle and cellular Apoptosis. A. The effects of NMN on the GC cell cycle. B. The effects of NMN on the GC cellular apoptosis.

Table S1 Immune checkpoint genes for immunotherapy responses in the risk groups

Table S2 Primers sequence for QPCR

Table S3 The correlation between NMRGs and lncRNAs

Table S4 Immuegene different expression between high-and low- risk groups

Table S5 The correlation between lncRNA and immune checkpoint genes

## Data Availability

The TCGA-STAD datasets (stomach adenocarcinoma, STAD), including gene expression profiles, DNA mutations, and clinic information, were obtained from GDC (https://portal.gdc.cancer.gov/cart). All analyzed data are included in this published article and its supplementary information file. Further inquiries can be directed to the corresponding authors.

## Supplementary Materials

The supplementary material is available online at https://doi.org/10.32604/or.2023.044618.
