# Supplementary figures and images for: NAD+ associated genes as potential biomarkers for predicting the prognosis of gastric cancer
Source: Oncol Res. 2023 Dec 28;32(2):283–96. doi: 10.32604/or.2023.044618 (PMC10765132; doi:10.32604/or.2023.044618)

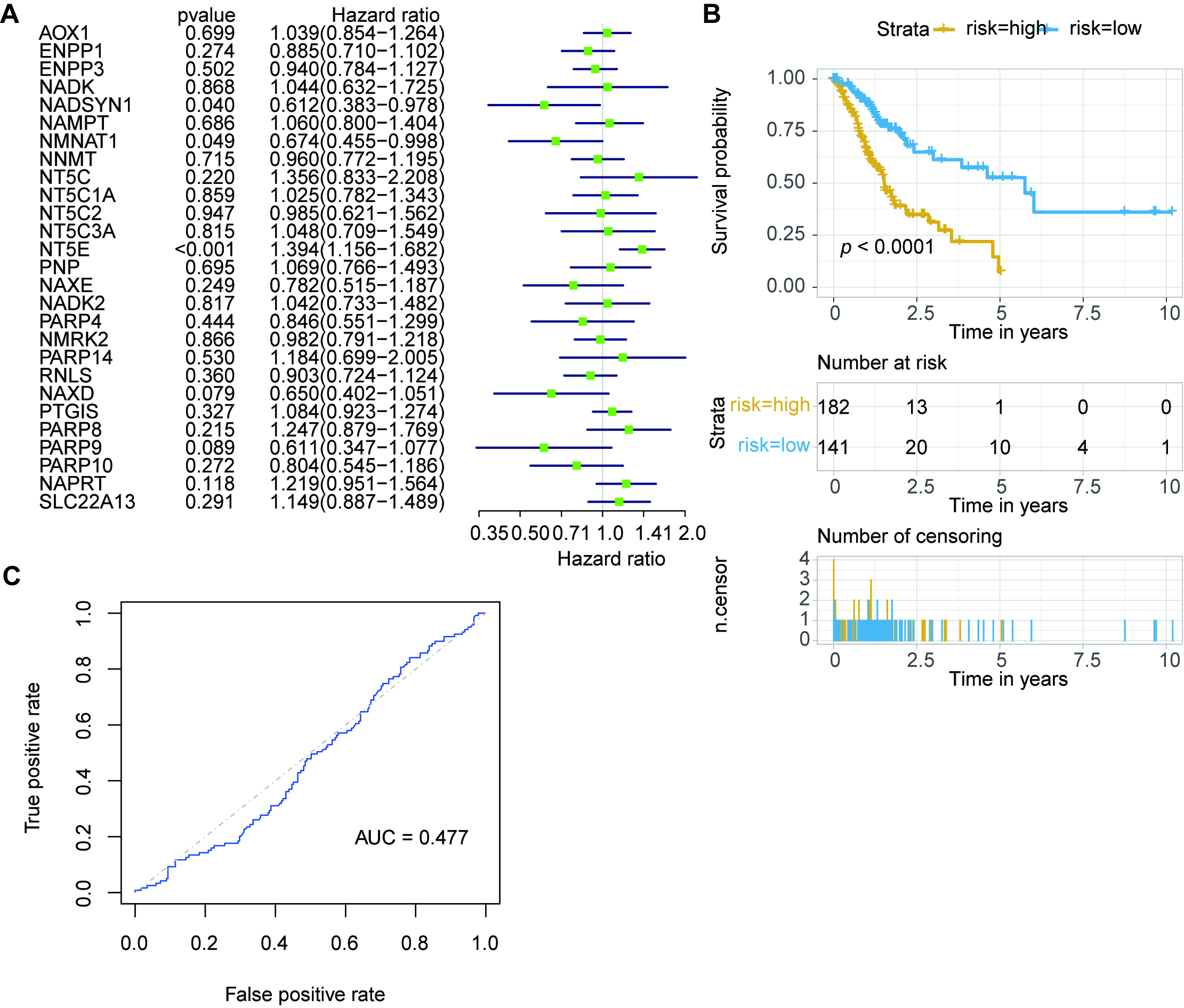

Supplement: Figure S1 [file OncolRes-32-44618-s001.tif]

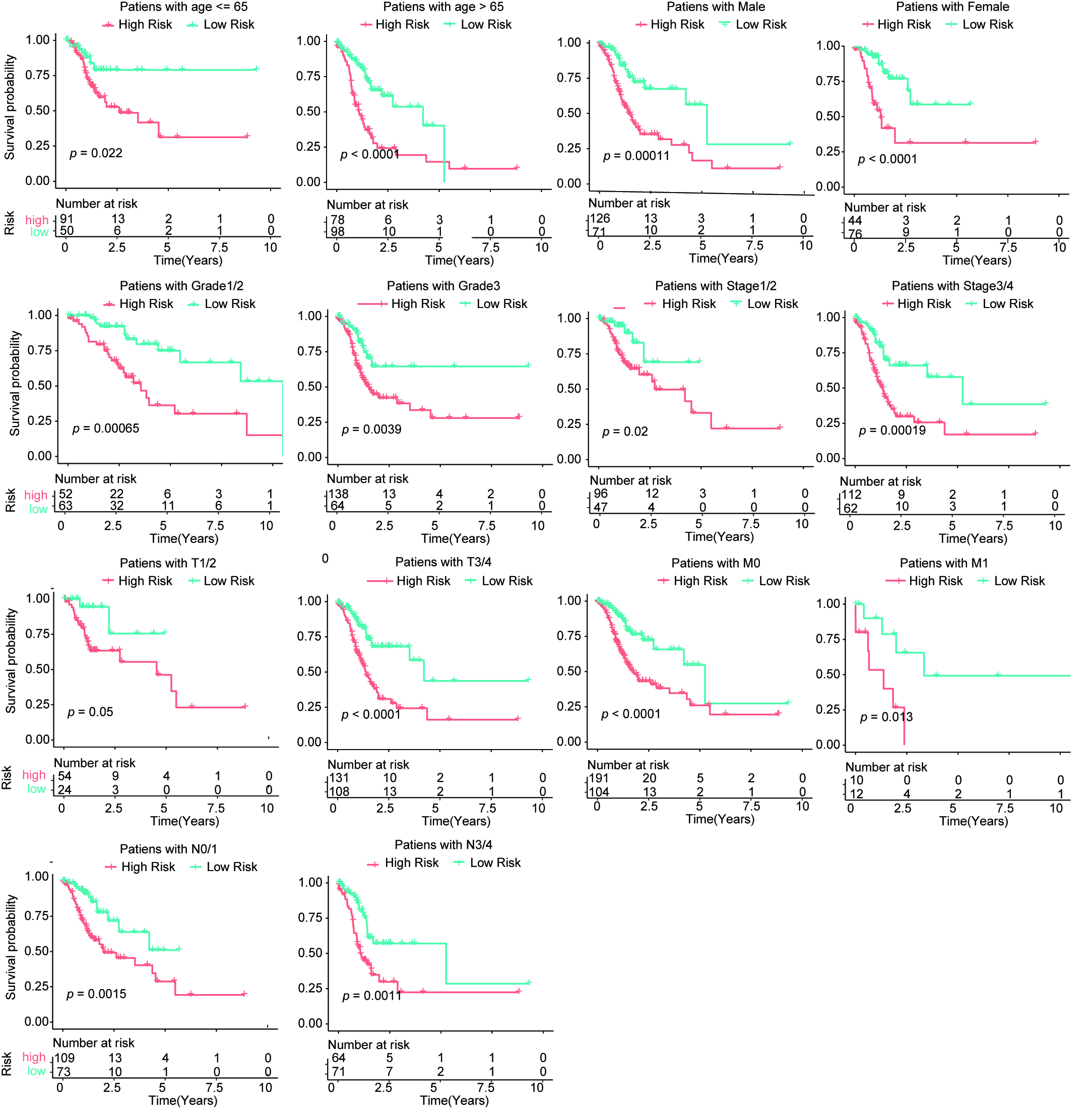

Supplement: Figure S2 [file OncolRes-32-44618-s002.tif]

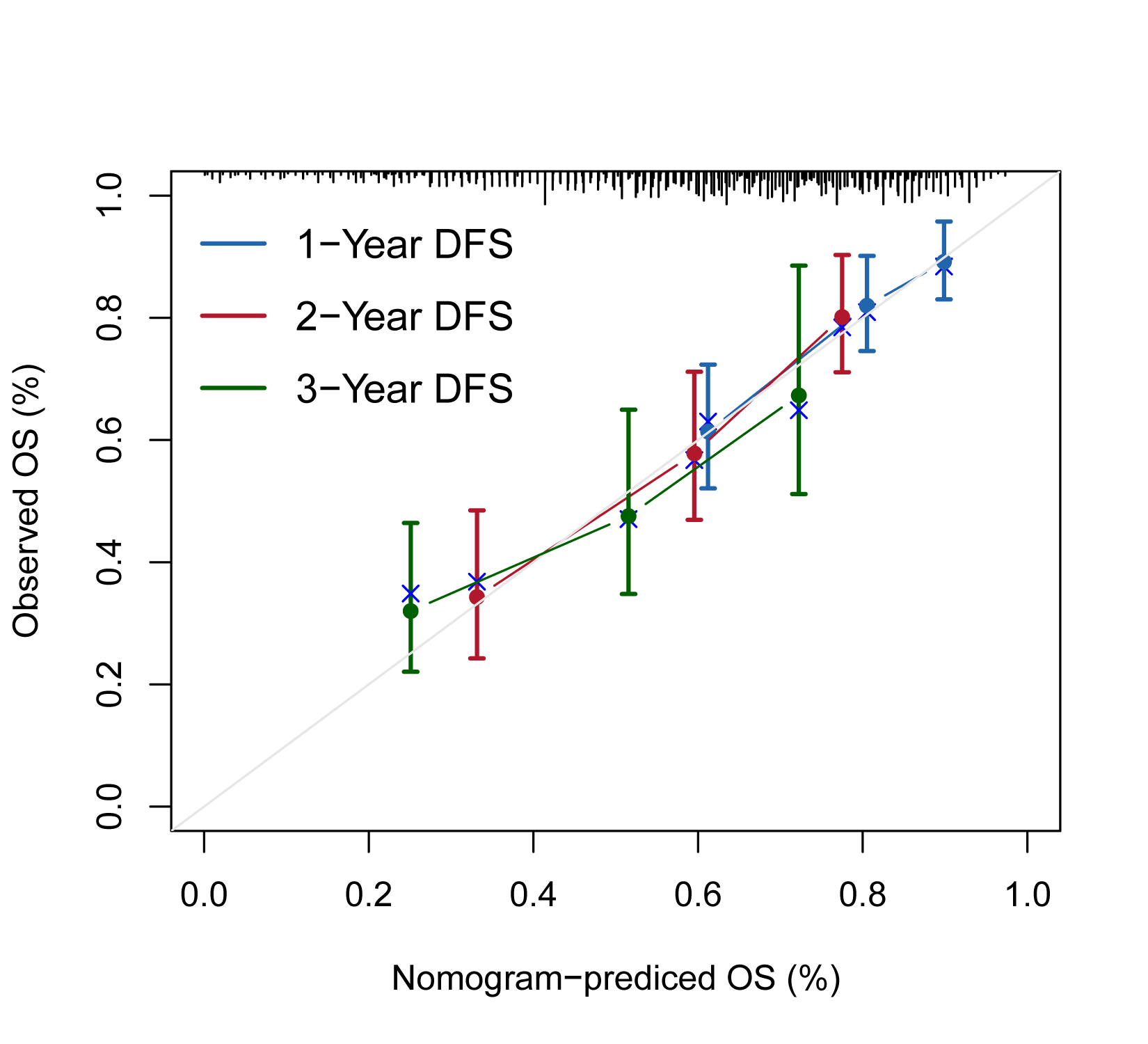

Supplement: Figure S3 [file OncolRes-32-44618-s003.tif]

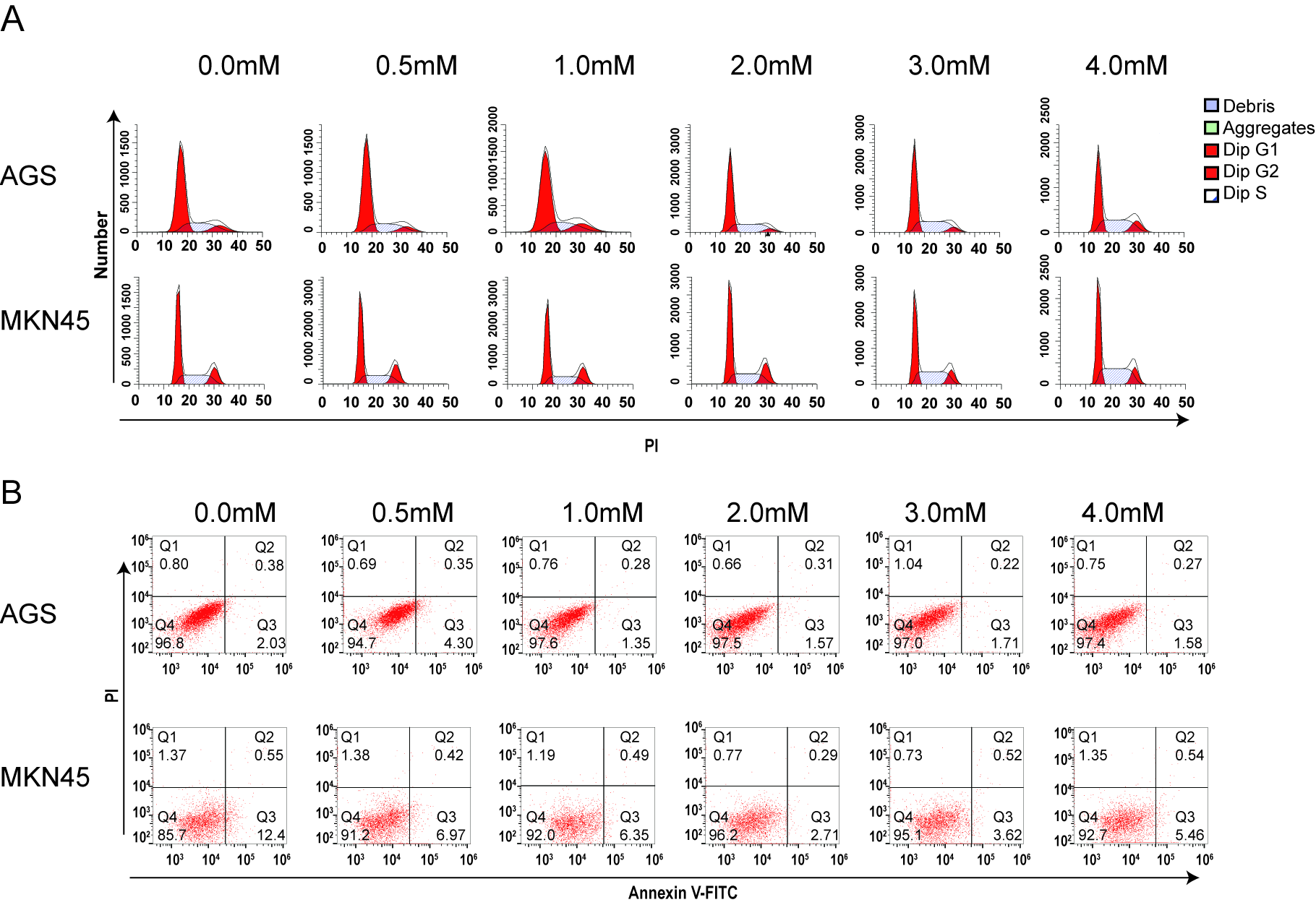

Supplement: Figure S4 [file OncolRes-32-44618-s004.tif]
